# Multipotent fetal stem cells in reproductive biology research

**DOI:** 10.1186/s13287-023-03379-4

**Published:** 2023-06-07

**Authors:** Margit Rosner, Stefanie Horer, Michael Feichtinger, Markus Hengstschläger

**Affiliations:** 1 Institute of Medical Genetics, Center for Pathobiochemistry and Genetics, Medical University of Vienna, Währinger Strasse 10, 1090 Vienna, Austria; 2 Wunschbaby Institut Feichtinger, Lainzerstraße 6, Vienna, Austria

**Keywords:** Fetal stem cells, Multipotency, Fetomaternal microchimerism, Germ cells, Gametogenesis, Reproductive system disease, Stem cell therapy

## Abstract

Due to the limited accessibility of the in vivo situation, the scarcity of the human tissue, legal constraints, and ethical considerations, the underlying molecular mechanisms of disorders, such as preeclampsia, the pathological consequences of fetomaternal microchimerism, or infertility, are still not fully understood. And although substantial progress has already been made, the therapeutic strategies for reproductive system diseases are still facing limitations. In the recent years, it became more and more evident that stem cells are powerful tools for basic research in human reproduction and stem cell-based approaches moved into the center of endeavors to establish new clinical concepts. Multipotent fetal stem cells derived from the amniotic fluid, amniotic membrane, chorion leave, Wharton´s jelly, or placenta came to the fore because they are easy to acquire, are not associated with ethical concerns or covered by strict legal restrictions, and can be banked for autologous utilization later in life. Compared to adult stem cells, they exhibit a significantly higher differentiation potential and are much easier to propagate in vitro. Compared to pluripotent stem cells, they harbor less mutations, are not tumorigenic, and exhibit low immunogenicity. Studies on multipotent fetal stem cells can be invaluable to gain knowledge on the development of dysfunctional fetal cell types, to characterize the fetal stem cells migrating into the body of a pregnant woman in the context of fetomaternal microchimerism, and to obtain a more comprehensive picture of germ cell development in the course of in vitro differentiation experiments. The in vivo transplantation of fetal stem cells or their paracrine factors can mediate therapeutic effects in preeclampsia and can restore reproductive organ functions. Together with the use of fetal stem cell-derived gametes, such strategies could once help individuals, who do not develop functional gametes, to conceive genetically related children. Although there is still a long way to go, these developments regarding the usage of multipotent fetal stem cells in the clinic should continuously be accompanied by a wide and detailed ethical discussion.

## Introduction

### Definition and classification of stem cells

**Fig. 1:**
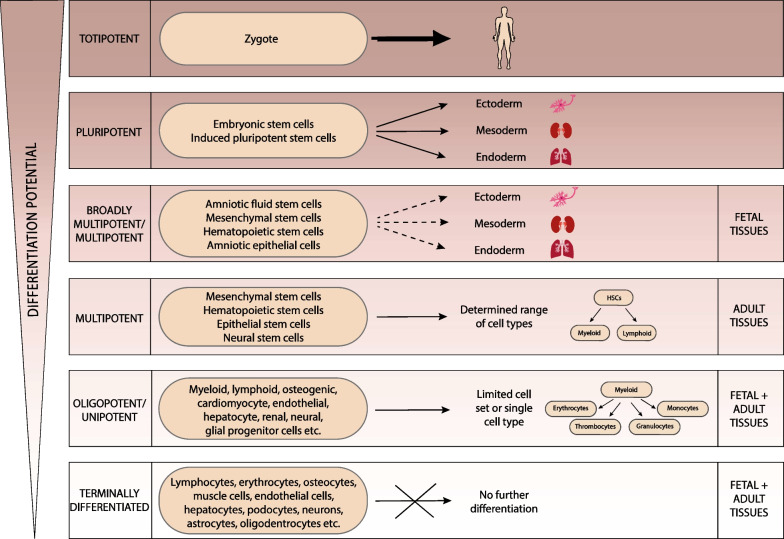
From totipotent to terminally differentiated cells. Classification of cells according to their differentiation potential. For details see the text. (All the images depicted in the figures of this report have been generated by the authors of this report.)

Stem cells are not-terminally committed cells making use of asymmetric cell division to differentiate and to self-renew maintaining their cellular identity in the course of proliferation [1]. Classifications of stem cells are typically based on their origin or differentiation potential [2]. With regard to their potential, the top of the hierarchy is formed by totipotent cells, which can differentiate into all cell types including placenta cells. Human embryogenesis starts with the totipotent zygote resulting from an oocyte fertilized by a spermatozoon, which then develops into the blastocyst. The blastocyst consists of the trophoblast, which gives rise to most of the placenta, and the inner cell mass, which develops into the embryo proper upon implantation into the uterine tissue [3]. The next level below is represented by pluripotent stem cells (Fig. 1). Both, embryonic stem cells (ESCs), derived from the inner cell mass of the blastocyst, and laboratory-made induced pluripotent stem cells (iPSCs), express pluripotency markers, exhibit indefinite self-renewal and can differentiate into cells of all three embryonic germ layers: ectoderm, mesoderm, and endoderm. The ectodermal lineage gives rise to, e.g., neural tissues, mesoderm develops, e.g., into renal, hematopoietic, endothelial or osteogenic tissues, and the endoderm lineage gives rise to, e.g., lung epithelial and hepatic tissues. Whereas iPSCs generated via reprogramming of somatic cells do not raise the ethical concerns regarding the moral status of the embryo, both pluripotent stem cell types share inherent tumorigenicity. Actually, for human stem cells, the proof of a teratoma formation upon injection into immunodeficient mice is common practice to confirm their functional pluripotency [4, 5]. Since significant interspecies differences impede the direct translation of results obtained with animal model organisms, human ESCs and iPSCs became increasingly relevant for research on human development and pathologies. Furthermore, although there are still the hurdles of immunogenicity and tumorigenicity to cross, human ESCs and iPSCs are well under way to their safe clinical translation in the course of a variety of innovative therapeutic concepts [6–9]. In addition, for other cells, distinct properties have been suggested. For example, very small embryonic-like stem cells (VSELs) have been discussed to be embryonic-like, to be pluripotent, to originate from cells related to the germline, and to also resident in adult bone marrow, peripheral blood, and various adult organs [10, 11].

So-called multipotent stem cells exhibit a more limited plasticity than pluripotent stem cells and can only differentiate into a determined range of cell types. Adult multipotent stem cells can be found in almost all tissues including lung, muscle, adipose tissue, bone marrow, skin, and even brain. Well characterized specimens of this group are mesenchymal stem cells (MSCs), harboring the potential to differentiate into a limited set of cells of the mesodermal, endodermal and ectodermal lineages; hematopoietic stem cells (HSCs) developing into lymphoid or myeloid cell types; epithelial stem cells (EpSCs), giving rise to, e.g., keratinocytes, hair follicles, or specific glands; and neural stem cells (NSCs), which can differentiate into astrocytes, oligodendrocytes or neurons [7, 8, 12]. Importantly, in the literature, the abbreviation “MSCs” is also used to describe mesenchymal stromal cells. Whereas mesenchymal stem cells are characterized by self-renewal and the potential to differentiate into a determined range of mesodermal, ectodermal or endodermal cell types, mesenchymal stromal are defined as a plastic adherent cell entity, expressing a defined mesenchymal marker spectrum, exhibiting specific immunomodulatory, secretory and homing properties and harboring a mesodermal differentiation potential giving rise to chondrocyte, osteoblast and adipocyte lineages [12–14].

Whenever cells can only differentiate into a very limited set of cells or even only into one single cell type, they are often designated oligopotent or unipotent “progenitors” rather than “stem cells”. In contrast to pluri- and multipotent stem cells, progenitor cells do not necessarily harbor an unlimited self-renewal ability. In Fig. 1, the example of HSCs and their descendant progenitors is graphically depicted. HSCs evolve into myeloid and lymphoid progenitor cells. Myeloid progenitors, having lost the lymphoid differentiation potential to develop into lymphocytes and natural killer cells, can only differentiate into cells of the myeloid lineage, such as, e.g., erythrocytes, thrombocytes, granulocytes, or monocytes. Beside myeloid and lymphoid progenitors, many more human cell types are known to function as origins of the development into terminally differentiated cells (Fig. 1) [1, 2, 4, 6, 8].

### The intermediate status of multipotent fetal stem cells

In the recent past, a specific group of stem cells entered the fore, for which differing nomenclature is used. At the outset, it is important to note that both commonly used designations, “fetal stem cells” and “perinatal stem cells”, do not reflect statements concerning the potential of these cells. In the context of the work on this review, we preferred the terminus “fetal” to “perinatal” for several reasons: 1) “Fetal” delineates both, the fetal origin of these cells and the time span of collection, whereas “perinatal” is exclusively related to a specific time period and can also include maternal cells. 2) According to the World Health Organization, the fetal period spans the time from the 9th week of gestation until birth and the perinatal interval refers to the period before and after birth, between 22 weeks after fertilization and 7 days after parturition (https://icd.who.int). Actually, in the context of stem cell research, the designation “perinatal” is mostly used to describe birth-associated tissues obtained from term placentas and fetal annexes [15]. Almost all knowledge on a very potent fetal stem cell entity, the so-called amniotic fluid stem cells (AFSCs), is derived from studies upon amniocenteses usually performed around the 16^th^ week of pregnancy and can therefore not be assigned to the perinatal period [16, 17]. 3) The designation “perinatal stem cells” also captures non-fetal stem cells, such as, e.g., MSCs from the decidua parietalis, a maternal component of perinatal tissues [15, 18–20]. In the here presented deliberations, we wanted to focus on stem cells of fetal origin, which can be banked to be deployed in autologous stem cell therapies later in life. Furthermore, fetal cells are considered to bear less accumulated mutations, what is of high relevance in the context of the reliability of basic research results and for the application of stem cell-based therapies in reproductive biology.

In compliance with the locally applicable guidelines for fetal tissue research and upon institutional ethical approval, fetal stem cells can also be isolated from surplus fetal tissues after first- or second-trimester termination of pregnancy [21]. Stem cells have been described to reside in, e.g., fetal liver [22–24], fetal lung [25], fetal pancreas [26, 27], or fetal kidney [28]. However, these fetal stem cell types are not easily accessible, raise several ethical issues, occur in small numbers and also exhibit only limited differentiation potential [21, 29, 30].

**Fig. 2:**
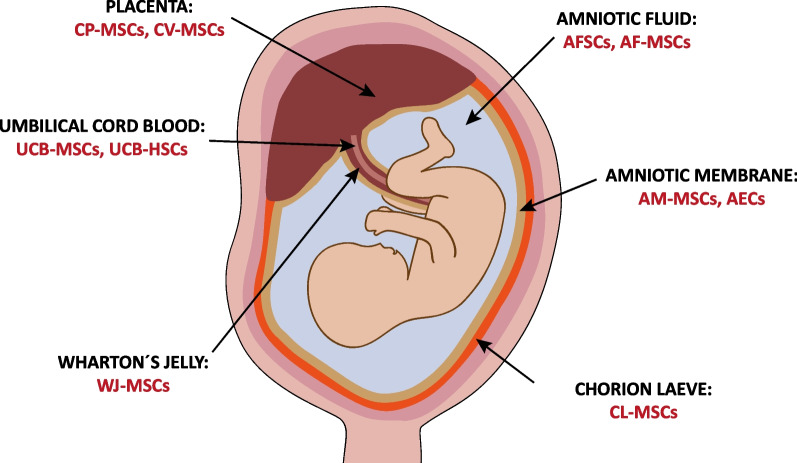
The different sources of multipotent fetal stem cells. AFSCs, c-Kit + amniotic fluid stem cells; AF-MSCs, amniotic fluid mesenchymal stem cells; AM-MSCs, amniotic membrane mesenchymal stem cells; AECs, amniotic epithelial cells; CL-MSCs, chorion laeve mesenchymal stem cells; WJ-MSCs, Wharton’s jelly mesenchymal stem cells; UCB-MSCs, umbilical cord blood mesenchymal stem cells; UCB-HSCs, umbilical cord blood hematopoietic stem cells; CP-MSCs, chorionic plate mesenchymal stem cells; CV-MSCs, chorionic villi mesenchymal stem cells

Primarily due to their broad differentiation potential, their high proliferation rate, and their low immunogenicity a specific set of fetal stem cell entities moved into the focus of today’s research interest: c-Kit + AFSCs (from the amniotic fluid); MSCs derived from the chorionic plate (CP-MSCs) or the chorionic villi (CV-MSCs) of the placenta, from amniotic fluid (AF-MSCs), the amniotic membrane (AM-MSCs), chorion laeve (CL-MSCs), Wharton´s jelly (WJ-MSCs), and umbilical cord blood (UCB-MSCs); HSCs from umbilical cord (UCB-HSCs); and amniotic epithelial cells (AECs) (Fig. 2). The fact that these stem cells are easy to sample without ethical controversies additionally underscores their outstanding role as highly valuable candidates for basic research and clinical applications. Although AFSCs and AF-MSCs are predominantly collected upon elective amniocentesis, all these stem cell types are accessible via non-invasive procedures. In fact, all these tissues, typically considered medical waste, would otherwise be discarded at birth [30–33]. Furthermore, compared to stem cells from adult tissues, these fetal stem cell types proliferate faster in culture, harbor higher tolerogenic properties, and many of them exhibit a greater differentiation potential. A significant proportion of these fetal stem cell entities can consistently be cultivated in vitro, are not tumorigenic and harbor the potential to differentiate into many cell types of all three embryonic germ layers. The latter granted them the designation “broadly multipotent” and the assignment of a place in the spectrum between pluripotent and multipotent stem cells. With the exception of AF-MSCs, CP-MSCs, and umbilical cord blood-derived cells, the here discussed fetal stem cells exhibit markers and features of both, multipotency and pluripotency, what does not necessarily imply that they can develop into every type of tissue. Broadly multipotent fetal stem cells are developmentally and operationally located between ESCs/iPSCs and adult stem cells (Figs. 1 and 2) [33–38].

In this review, we discuss the recent advancements in this field with the endeavor to shed more light on the following questions: 1) What are the various characteristics of the different multipotent fetal stem cell types? 2) What is there biological role? 3) How can they be used for basic research in reproductive biology? 4) What are the currently pursued research strategies to drive their clinical application for reproductive system diseases?

## Characteristics of the different multipotent fetal stem cell entities

### c-Kit + amniotic fluid stem cells

To distinguish AFSCs from other stem cells and progenitors floating in the amniotic fluid, the term “amniotic fluid stem cells” should exclusively be used for broadly multipotent Oct4-expressing stem cells isolated from amniotic fluid by immunoselection for c-Kit (CD117), the receptor for stem cell factor (SCF) [39]. This fetal stem cell entity discovered in 2003 [16] expresses several pluripotency markers and exhibits self-renewal capacity in the course of non-adhesive in vitro proliferation over multiple passages without signs of genomic instability [17, 40]. Studies using monoclonal lines demonstrated that AFSCs can form embryoid bodies [41] and differentiate into cells of all three embryonic germ layers, but do not form teratomas when transplanted into immunodeficient mice [17]. c-Kit + AFSCs have been demonstrated to share 82% transcriptome identity with ESCs and can be programmed to full functional pluripotency including the ability to form teratomas upon injection into immunodeficient mice merely by treatment with the histone deacetylase inhibitor valproic acid [42] (Figs. 1 and 2, Table 1).

**Table 1:** Characterization of the different types of multipotent fetal stem cells

Stem cells	Cell morphology	Markers positive	Markers negative	MHC class expression	Differentiation potential	Tera- toma	Secreted factors	References
*Amniotic fluid*								
AFSCs	“Embryonal”, mesenchymal	c-Kit (CD117), Oct4, c-Myc, Rex1, SSEA4, SDF1-receptor, CXCR4, CD29, CD44, CD73, CD90, CD105, CD146, CD166, CD184	Klf4, Nanog, ALP, Sox2, SSEA1, SSEA3, Tra-1–60, Tra-1-81, CD34, CD80, CD86, CD133	MHC I + MHC II-	Ecto-, Meso-, Endoderm	No	IL-6, IL-8, VEGF, Tβ4, SDF-1, MCP-1, IGF-I, IGF-II	[16, 17, 82, 93–95, 183, 184]
AF-MSCs	Mesenchymal	CD29, CD44, CD73, CD90, CD105	c-Kit (CD117), CD31, CD34, CD45	MHC I + MHC II-	Ecto- and Mesoderm	Not tested	IL-8, VEGF, EGF, TGFβ, TNFR1	[49, 50, 96, 185]
*Amniotic membrane*								
AM-MSCs	Mesenchymal	Oct4, GATA4, Klf4, Nanog, Sox7, Sox17, FOXC1, TBX6, SSEA3, SSEA4, CD10, CD13, CD24, CD29, CD44, CD49e, CD54, CD73, CD90, CD105, CD166	c-Kit (CD117), Rex1, Tra-1-60, Tra-1-81, CD14, CD19, CD31, CD34, CD45	MHC I ± MHC II-	Ecto-, Meso-, Endoderm	Not tested	IL-6, IL-8, PDGF, VEGF, TGFβ, TGFβ2, IGF-1, HGF, G-CSF, GM-CSF, TIMP1, TIMP2, bFGF, TNFα, MIP1α, MIP1β, Ang-1, RANTES, PGE2, VCAM-1, Oncostatin M, Angiogenin	[51–53, 56, 187–190,186]
AECs	Epithelial	Oct4, Rex1, Nanog, GATA4, Cripto, SSEA3, SSEA4, Sox2, Tra-1-60, Tra-1-81, CD9, CD10, CD13, CD24, CD29, CD44, CD49e, CD73, CD90, CD105, CD166	c-Kit (CD117) (no/very low expression), SSEA1, CD14, CD31, CD34, CD45, CD49d, CD79, CD133	MHC I + MHC II ±	Ecto-, Meso-, Endoderm	No	IL-6, IL-8, IL-1ra, VEGF, TGFβ, TGFβ2, TIMP1, TIMP2, PGE2, TNFα, MIF, GM-CSF, G-CSF, PDGF, bFGF, MIP1α, MIP1β, RANTES, Oncostatin M, Angiogenin	[53, 67–70, 186–189, 191]
*Chorion laeve*								
CL-MSCs	Mesenchymal	Oct4, Rex1, Nanog, GATA2, Sox7, Sox17, FOXC1, TBX6, SSEA3, SSEA4, Tra-1-60, Tra-1-81, CD13, CD19, CD29, CD44, CD54, CD73, CD90, CD105, CD166	c-Kit (CD117), CD3, CD14, CD19, CD31, CD34, CD45	MHC I + MHC II-	Ecto-, Meso-, Endoderm	Not tested	IGF-1, VEGF, TGF, HGF, bFGF, Ang-1; shown for human amnion/chorion membrane: PDGF-AA, TGFβ1, bFGF	[54–56, 98–100, 189, 192–197]
*Wharton´s jelly*								
WJ-MSCs	Mesenchymal	Oct4, Klf4, Nanog, Sox2, SSEA3, SSEA4, c-Myc, GFAP, MBP, MAP-2, nestin, CD10, CD13, CD29, CD44, CD73, CD90, CD105, CD146, CD166	CD11, CD14, CD19, CD31, CD34, CD38, CD40, CD45, CD80, CD86, CD106, CD133	MHC I + MHC II-	Ecto-, Meso-, Endoderm	Not tested	IL-1α, IL-6, IL8, IL-17, IGFBPs, ICAM-1, VCAM-1, HGF, SCF, MCP-1, Serpins, CXCL5, Ang-1, Endostatin, aFGF, LAP, MMP-9, IGF-1, VEGF, TGFβ1, NRG1-B1, Persephin, Prolactin, PGE2, Angiogenin, Platelet factor 4	[58, 59, 97, 101–103, 136, 198–205]
*Umbilical cord blood*								
UCB-MSCs	Mesenchymal	CD13, CD29, CD44, CD51, CD58, CD71, CD73, CD90, CD105, CD146, CD166	c-Kit (CD117), CD14, CD19, CD31, CD33, CD34, CD45, CD51, CD64, CD106, CD133, CD135	MHC I + MHC II +	Mesoderm	Not tested	IL-6, IL-8, G-CSF, CXCL1, PAI-1, MIF, MCP-1	[57, 104, 105, 206–208]
UCB-HSCs	Hematopoietic	c-Kit (CD117), CD34, CD45, CD71, CD90, CD95, CD133, CD135	CD2, CD3, CD4, CD5, CD7, CD8, CD10, CD14, CD15, CD16, CD19, CD20, CD24, CD33, CD38, CD41, CD56, CD66b, CD71	MHC I + MHC II +	Myeloid and lymphoid	Not tested		[37, 64–66]
*Placenta*								
CP-MSCs	Mesenchymal	CD13, CD44, CD54, CD56, CD71, CD73, CD90, CD95, CD105, CD106, CD166	CD14, CD19, CD31, CD33, CD34, CD45, CD51	MHC I + MHC II-	Meso- and Endoderm	Not tested	Ang-1, HGF, IGF-1, TGF-β1, VCAM-1, PGE2	[45, 46, 48, 97, 106, 209]
CV-MSCs	Mesenchymal	c-Kit (CD117); Oct 4/Nanog (1^st^ trimester), Sox2, SSEA4, CXCR4, CD11a, CD13, CD29, CD44, CD49b,d,e,f, CD51, CD73, CD90, CD105, CD106, CD166	Oct4/Nanog (at term), CD14, CD19, CD34, CD45, CD56, CD80, CD83, CD86	MHC I + MHC II-	Ecto-, Meso-, Endoderm	Not tested	IL-1α + β, IL-8, HGF, PDGF-BB, GM-CSF, G-CSF, CXCL1, Ang-2, MCP-3, TARC, RANTES, OPG, μPAR, CTACK	[43, 44, 47, 107, 133, 135, 210–214]

Marker descriptions and stem cell features are included in this table when they are documented by several publications

AFSCs, c-Kit + amniotic fluid stem cells; AF-MSCs, amniotic fluid mesenchymal stem cells; AM-MSCs, amniotic membrane mesenchymal stem cells; AECs, amniotic epithelial cells; CL-MSCs, chorion laeve mesenchymal stem cells; WJ-MSCs, Wharton’s jelly mesenchymal stem cells; UCB-MSCs, umbilical cord blood mesenchymal stem cells; UCB-HSCs, umbilical cord blood hematopoietic stem cells; CP-MSCs, chorionic plate mesenchymal stem cells; CV-MSCs, chorionic villi mesenchymal stem cells

ALP, alkaline phosphatase; Ang, angiopoietin; aFGF, acidic fibroblast growth factor; bFGF, basic fibroblast growth factor; CD, cluster of differentiation; c-Kit, tyrosine-protein kinase Kit (receptor for SCF); c-Myc, cellular myelocytomatosis oncogene product; Cripto, epidermal growth factor-like Cripto protein CR1; CTACK, cutaneous T-cell-attracting chemokine; CXCR4, C-X-C Motif Chemokine Receptor 4, SDF-1-receptor; CXCL, C-X-C motif chemokine ligand; EGF, epidermal growth factor; FOXC1, Forkhead box C1 protein; GATA, GATA-binding protein; G-CSF, granulocyte-colony stimulating factor; GFAP, glial fibrillary acidic protein; GM-CSF, granulocyte macrophage-colony stimulating factor; HGF, hepatocyte growth factor; ICAM-1, intercellular adhesion molecule-1; IGF, insulin-like growth factor; IGFBP, insulin-like growth factor-binding protein; IL, interleukin; IL-1ra, interleukin 1 receptor antagonist; Klf4, Krüppel-like factor 4; LAP, latency-associated peptide; MAP-2, microtubule-associated protein-2; MBP, myelin basic protein; MCP, monocyte chemoattractant protein; MHC, major histocompatibility complex; MIF, macrophage migration inhibitory factor; MIP, macrophage inflammatory proteins; MMP, matrix metalloproteinase; Nanog, homeobox protein Nanog; NRG1-B1, neuregulin-1-B1; Oct4, octamer-binding transcription factor 4; OPG, osteoprotegerin; PAI-1, plasminogen activator inhibitor-1; μPAR, urokinase plasminogen activator receptor; PDGF, platelet-derived growth factor; PGE2, prostaglandin E2; RANTES, regulated on activation, normal T cell expressed and secreted = chemokine (C-C motif) ligand 5 (CCL5); Rex1, redox-sensing transcriptional repressor Rex1; SCF, stem cell factor; SDF-1, stromal cell-derived factor 1; Sox, SRY-box transcription factor; SSEA, stage specific embryonic antigen; Tβ4, Thymosin β4; TBX6, T-Box transcription factor 6; TARC, thymus- and activation-regulated chemokine; TGF, transforming growth factor; TIMP, tissue inhibitor of metalloproteinases; TNF, tumor necrosis factor; TNFR1, tumor necrosis factor receptor 1; Tra-1-60 and Tra-1-81, antibodies recognizing epitopes on podocalyxin; VCAM1, vascular cell adhesion molecule 1; VEGF, vascular endothelial growth factor

### Mesenchymal stem cells in the placenta, amniotic fluid, amniotic membrane, chorion laeve, Wharton´s jelly, and umbilical cord blood

**Fig. 3:**
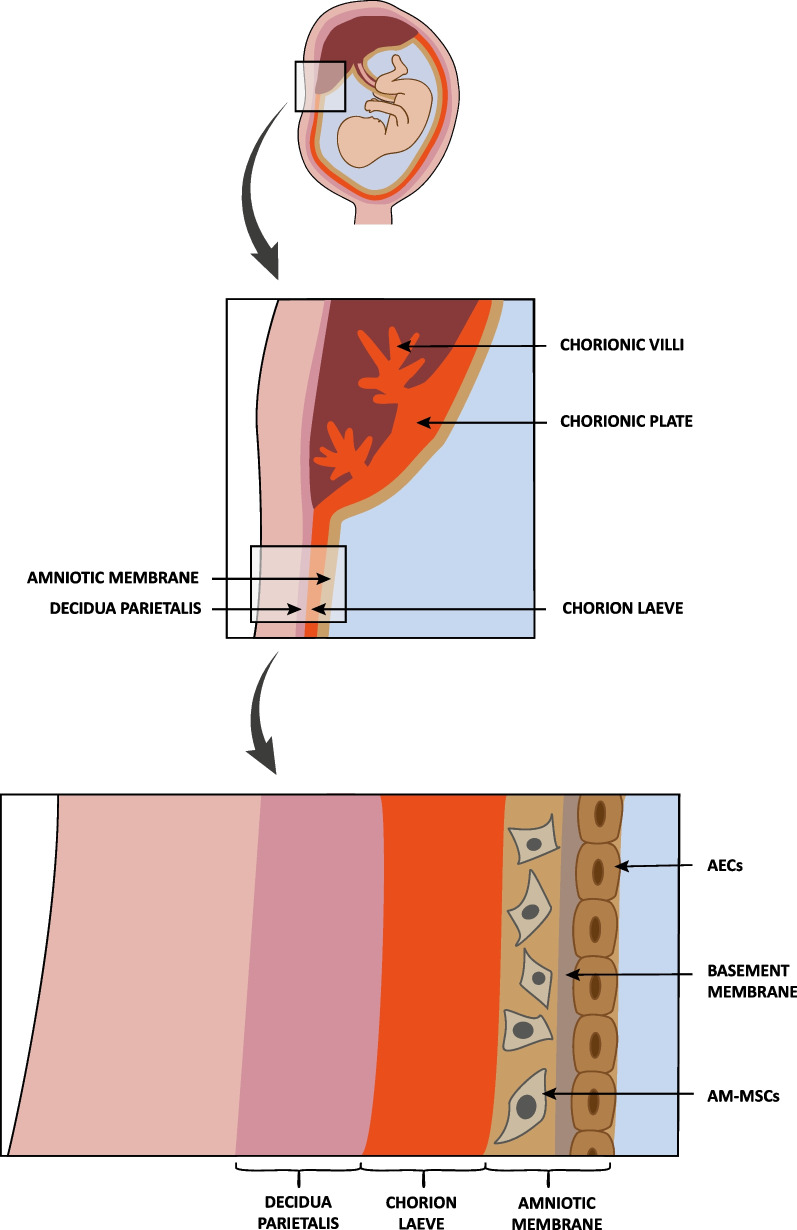
The placenta and fetal membranes as sources of multipotent fetal stem cells. Enlarged schematic views of the placenta with focus on the chorionic plate and chorionic villi and of the extra-embryonic membranes: the maternal decidua parietalis, the chorion laeve, and the amniotic membrane consisting of the outer layer with amniotic membrane mesenchymal stem cells (AM-MSCs), the cell-free basement membrane and the inner layer of amniotic epithelial cells (AECs)

Two different components of the placenta have been identified as rich sources for fetal MSCs: the chorionic plate, containing the fetal part of the placental disk, and the chorionic villi, which are projections sprouting from the chorion and reaching from the chorionic plate into the intervillous space to provide maximal contact with the maternal blood (Figs. 2 and 3, Table 1) [32, 43–48]. Beside the c-Kit + AFSCs described above, amniotic fluid contains another fetal stem cell type, the less widely explored AF-MSCs, which are negative for c-Kit (CD117) (Fig. 2, Table 1) [39, 49, 50]. The innermost component of the fetal membranes is the amniotic membrane (amnion) which is the inner layer of the amniotic sac consisting of an epithelial monolayer composed of AECs, an acellular basement membrane, and a mesenchymal cell layer built up by AM-MSCs (Figs. 2 and 3) [15, 34, 51–53]. The next layer attached to the amniotic membrane (and in close contact to the maternal decidua parietalis) is designated chorion laeve. The chorion laeve, also called smooth chorion, belongs to the chorionic membrane (chorion), but is in contrast to the chorionic plate and the chorionic villi not involved in the formation of the definitive placenta (Figs. 2 and 3) [15, 34]. The chorion laeve has been demonstrated to be a rich source for multipotent fetal CL-MSCs [54–56]. The umbilical cord connects the fetus with the placenta to ensure the continuous supply of nutrients and oxygen to the unborn child. It contains one umbilical vein and two umbilical arteries surrounded by a mucoid connective tissue designated Wharton’s jelly. MSCs can be isolated from both sources, the Wharton’s jelly and the umbilical cord blood (Fig. 2, Table 1) [32, 38, 57–59].

The International Society for Cellular Therapy initiated a discussion about the characteristics, which must be fulfilled to identify a cell as a MSCs [13, 14, 18]. Without allowing definitive conclusions regarding the stemness (self-renewal, differentiation potential etc.) of a cell, the lack of cell surface molecules such as the hematopoietic markers CD34 and CD45 and the concurrent expression of CD73, CD90, and CD105 are considered to be elementary for a mesenchymal cell characterization. As presented in Table 1, all the here described fetal MSCs exhibit this spectrum of cell surface markers. However, regarding the co-expression of pluripotency markers, such as Oct4, Nanog, Sox2, Tra-1-60, Tra-1-81 and stage specific embryonic antigens (SSEAs), which are typically expressed by ESCs and iPSCs [37], these fetal MSCs differ significantly (Table 1). Furthermore, a difference has also been reported regarding the expression of the stem cell factor receptor c-Kit (CD117). And finally, whereas AM-MSCs, CL-MSCs, WJ-MSCs, and CV-MSCs harbor the potential to develop into cell types of all three embryonic germ layers, AF-MSCs, UCB-MSCs, and CP-MSCs have been described to exhibit limited differentiation potentials into ectoderm/mesoderm, mesoderm, and mesoderm/endoderm, respectively (see Table 1 and the reference cited therein).

### Hematopoietic stem cells in umbilical cord blood

UCB-HSCs, which have already been discovered several decades ago [60, 61], are less mature and harbor a higher self-renewal capacity than HSCs from adult sources [20, 62]. They exhibit long telomeres and a high telomerase activity [63] and are characterized by the expression of CD34 and c-Kit (CD117). Their multipotency is reflected by their capacity to differentiate into all cell types of the lymphoid or myeloid cell lineage (Table 1) [37, 64–66].

### Amniotic epithelial cells

Beside AM-MSCs, the amniotic membrane contains another cell type considered to exhibit stemness, the so-called AECs (also designated as amniotic membrane epithelial cells). AECs constitute the amniotic membrane epithelium, which is in touch with the amniotic fluid (Fig. 3). In addition to a typical mesenchymal spectrum of markers (positive for CD73, CD90, and CD105; negative for CD34, CD45), AECs also express the classical pluripotency markers Oct4, Nanog, Sox2, Tra-1-60, Tra-1-81, SSEA3, and SSEA4. Interestingly, it has been reported that AECs are either negative for c-Kit (CD117) or only a few cells express this marker at a very low level (Table 1) [53, 67–70]. Their expression of pluripotency markers as well as their self-renewal capacity, together with their potential to give rise to cells of all three germ layers suggested AECs to be a pluripotent stem cell entity. However, the observation that AECs do not form teratomas upon transplantation into immunodeficient mice formed the basis for their classification into “only” broadly multipotent stem cells [67, 70, 71].

## The biological role of multipotent fetal stem cells

Generally spoken, their self-renewal capacity and differentiation potential in cooperation with their anti-apoptotic, angiogenic, anti-inflammatory, and immunomodulatory properties enable stem cells to be involved in a variety of intercellular processes. Stem cells constitute the origin of cell and tissue development, regulate the functions of adjacent cells via paracrine signalling, form the promoting platform for tissue and organ regeneration, are stabilizers of the physiological functions of tissues and organs, and are thereby indispensable guardians of the intracorporeal homeostasis [7, 8, 12]. It is obvious that all these functions are of even higher relevance for fetal stem cells to fulfil their roles in the extremely dynamic processes of fetal development. The very progressive transformations affecting the fetal membranes, the umbilical cord, and the placenta during the fetal period must be initiated, supplied and constantly controlled. It is therefore not surprising that the stem cell entities, which are functionally engaged with these fetal tissues, must display self-renewal, a high differentiation potential, and eminent paracrine properties. In line with that, there is broad consensus that the remarkable properties of AM-MSCs, AECs, CL-MSCs, WJ-MSCs, UCB-MSCs, UCB-HSCs, CP-MSCs, and CV-MSCs essentially reflect their biological roles in the according tissues of the placenta and fetal annexes (Fig. 2, Table 1) [32–36, 38].

**Fig. 4:**
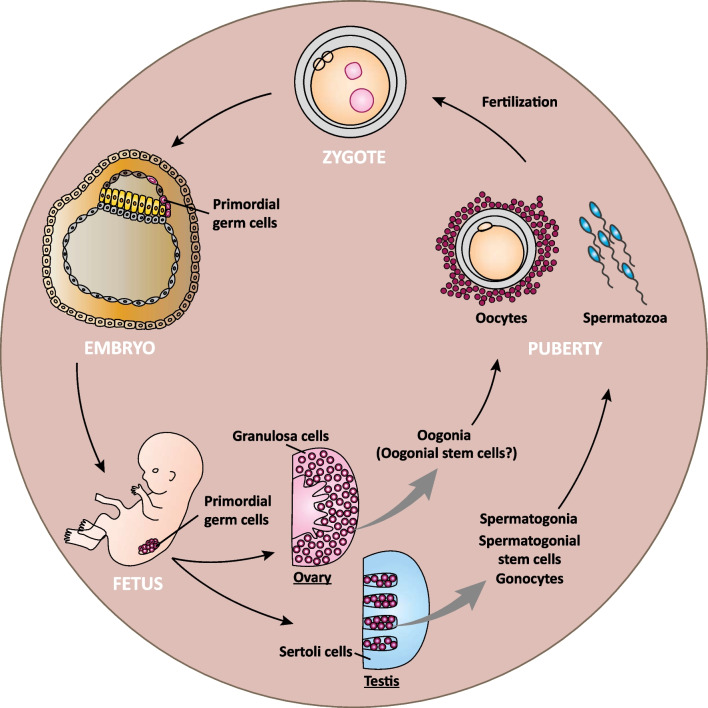
Germ cell development. Schematic illustration of the development of human diploid germ cells into the haploid gametes, the spermatozoa and oocytes

In this context, stem cells floating in the amniotic fluid and especially the Oct4- and cKit (CD117)-positive AFSCs are supposed to possess a unique status. To date, the origin of this broadly multipotent stem cell entity has not yet been discovered [39]. Already in the course of their first description, AFSCs have been speculated to be derived from aberrantly migrating PGCs (Fig. 4), which might have experienced specific alterations upon their migration from the tissue-specific microenvironment to the amniotic fluid [16]. But although AFSCs have been discussed to share some gene expressions characteristic of germ cells or PGCs [42, 72], the definitive proof of this hypothesis is still missing.

Furthermore, with regard to their biological role, increasing evidence supports the notion that their functional target tissues could be found in the mother rather than in the fetus or the extraembryonic tissues [73]. Already in the first weeks of pregnancy, fetal stem cells traffic into the maternal circulation. These fetal stem cells are considered to play a beneficial role for the mother mainly by their involvement in tissue regeneration. However, negative implications of this deposition of fetal cells in the mother’s body for the maternal health have also been discussed [74–76]. Although the first description of this phenomenon, designated fetomaternal microchimerism, goes back to the year 1893 [77], the origin of these fetal stem cells in the mother´s body still remains elusive [78, 79]. The so-called pregnancy-associated progenitor cells [80] are considered to be Oct4-positive, non-tumorigenic, broadly multipotent fetal stem cells, which can be mobilized from their fetal origin to exhibit paracrine effects on maternal cells and tissues. They should exhibit an anchorage-independent, long-lasting survival capacity, a non-adhesive proliferation potential, low immunogenicity, and genomic stability [74, 75, 81]. AFSCs fulfil all these criteria (Figs. 1 and 2, Table 1) [16, 17, 40, 41] and have also been shown to affect adjacent cells via paracrine signalling [82]. In 2017, animal experiments further demonstrated that they can be mobilized and recruited to injured maternal tissues upon injection into the amniotic fluid [83]. Whereas for the other here discussed fetal stem cells the different origins match their functional target tissues, the origin of AFSCs is still undetermined and although certain supportive evidence has been reported, the non-fetal sphere of AFSC activities also still awaits further investigation [39, 73].

## Multipotent fetal stem cells for basic research in reproductive biology

The specific features of the here discussed fetal stem cells highlight them as an optimal tool for basic research. First, the ease of their acquisition is one major reason for the wide application of these fetal stem cells as non-transformed, non-immortalized, primary cell models. The isolation of AFSCs, AF-MSCs, AM-MSCs, AECs, CL-MSCs, WJ-MSCs, UCB-MSCs, UCB-HSCs, CP-MSCs, and CV-MSCs is not associated with ethical objections, is covered by only minimal legal limitations, and their natural occurring multipotency is not the consequence of reprogramming approaches, which are associated with the risk to trigger mutations [2, 20, 30, 32, 38]. Furthermore, AFSCs, AF-MSCs, AM-MSCs, AECs, CL-MSCs, WJ-MSCs, UCB-MSCs, CP-MSCs, and CV-MSCs can be propagated under less demanding culture conditions as cell monolayers in a feeder-free, serum-rich environment. Under these conditions, they are genetically stable maintaining their euploid karyotype; they exhibit a high proliferation rate with a doubling time between 1 and 2 days, and show a high expansion potential without the tendency for spontaneous differentiation or early replicative senescence [15, 32, 38, 39]. In addition, fetal stem cells such as AFSCs can be used to establish monoclonal cell lines [17] to circumvent research studies performed on a mixture of different clonal variants. And importantly, the amenability of fetal stem cells for genetic modifications highlight them as a perfect tool for genetic, biochemical and cell biological basic research approaches [40, 82, 84–92].

Another characteristic of utmost importance for basic research is their wide differentiation potential (Table 1), which creates ideal conditions to study the underlying molecular mechanisms of various cell differentiation processes and allows the usage of the so obtained terminally differentiated cell types for investigations on the specific cellular entities. AFSCs can be differentiated into neural, renal, hematopoietic, adipogenic, myogenic, endothelial, chondrogenic, osteogenic, epithelial, and hepatic cell types [17, 39, 93–95]. AF-MSCs have been developed upon neural, adipogenic, and osteogenic lineages [49, 50, 96]. The differentiation potential of AM-MSCs includes the development into neural, myogenic, endothelial and hepatic cell types [51, 52, 95, 97] and AECs differentiate into neural, adipogenic, myogenic, osteogenic, and hepatic cells [53, 67, 70]. CL-MSCs can also develop into cells of all three embryonic germ layers including neural, adipogenic, chondrogenic, osteogenic as well as endodermal entities [54, 56, 98–100]. Whereas WJ-MSCs have been demonstrated to differentiate into neural, adipogenic, myogenic, endothelial, chondrogenic, osteogenic, and hepatic cell types [58, 59, 97, 101–103], the differentiation potential of UCB-MSCs is limited to mesodermal cell types such as adipogenic, chondrogenic, and osteogenic [57, 104, 105], and the hematopoietic UCB-HSCs exclusively differentiate into myeloid and lymphoid cells [64, 65]. CP-MSCs are known to harbor the potential to differentiate into adipogenic, chondrogenic, osteogenic and hepatogenic cell types [45, 46, 48, 106], and CV-MSCs can develop into neural, adipogenic, chondrogenic, osteogenic, and hepatic cells [43, 47, 107]. Taken together, all these established differentiation protocols are extremely valuable for molecular investigations on cell maturation processes. In this context, the usage of human fetal stem cells is of special interest because results obtained from model organisms cannot necessarily directly be assigned to humans. Due to the scarcity of human adult tissue material and the inaccessibility of the human in vivo condition, the in vitro differentiation of primary human fetal stem cells came more and more into focus.

This holds especially true for the use of multipotent fetal stem cells in the directed differentiation of germ cells, an already indispensable approach in today´s research on human reproduction. The in vitro differentiation of fetal stem cell-derived germ cells allows to study the underlying molecular basis of these processes with the hope to obtain a more comprehensive picture of pathologies affecting germ cell development [108]. Generally spoken, the generation of laboratory-made germ cell-derived gametes creates prospects for their future innovative use in medically assisted reproduction [109–112]. Accordingly, we particularly emphasized the experimental approach to use multipotent fetal stem cells for germ cell differentiation by a detailed presentation and discussion in the next chapter.

Another feature of the here debated fetal stem cells that provoked interest among the scientific community is their amenability for the transformation from a multipotent to a pluripotent state. The fact that compared to adult counterparts, fetal cells exhibit less naturally acquired somatic mutations makes them an attractive tool for the generation of iPSCs. Furthermore, starting from the level of broad multipotency reprogramming of fetal stem cells was assumed to allow the efficient generation of iPSCs with pluripotent features. Indeed, a variety of different protocols have been used to reprogram WJ-MSCs into iPSCs. The authors expressed their hope that at least part of the epigenetic signature representing the fetus could be retained in memory in the iPSCs derived from multipotent fetal stem cells [89, 90, 113]. Furthermore, human AM-MSCs have been reprogrammed to teratoma-forming iPSCs by ectopic expression of Oct4, Sox2, c-Myc, and Klf4 with high efficiency [84, 85]. The ectopic expression of the same combination of factors has been demonstrated to efficiently transform chorionic mesenchymal stromal cells obtained from term pregnancies [87, 114], UCB-HSCs [92, 115], as well as early second trimester AFSCs [116] into iPSCs. The latter stem cell entity has been found to be particularly susceptible to reprogramming into full functional pluripotency. It has been shown that human AFSCs with moderate endogenous expression of Oct4 can be reprogrammed into iPSCs by just one factor, the high level ectopic expression of Oct4. The so obtained iPSCs gain teratoma formation potential [88]. Remarkably, AFSCs can even be transformed into iPSCs without the expression of ectopic factors, just by cultivation on extracellular matrix in ESC medium upon incubation with the histone deacetylase inhibitor valproic acid [42, 95, 117].

Furthermore, fetal stem cells represent an important tool to obtain a more comprehensive picture of the underlying molecular processes of diseases originating from fetal tissues. For example, preeclampsia is caused by inadequate placentation, owing to deficient invasion of trophoblast cells into the lining of the uterus. This can result into placental hypoxia, abnormal expression of angiogenic factors, and oxygen deprivation in the embryo [118]. Another example is the condition of abnormal placental invasion known as placenta accreta, increta, and percreta, which can cause maternal morbidity and mortality [119].

And finally, research on fetal stem cells will pave the way to a better understanding of the advantageous and disadvantageous consequences of their migration into the maternal circulation during pregnancy. On the one hand, it is known that fetal stem cells can adopt the phenotype of maternal target tissues and contribute to organ regeneration. But on the other hand, these fetal microchimeric cells have also been demonstrated to harbor the potential to play a role in the development of maternal diseases. The molecular processes triggering these different effects can only be elucidated by further research on the cellular origins of fetomaternal microchimerism, including stem cells from the placenta and the amniotic fluid [39, 78, 120]. Next to fetal cell trafficking into the mother´s body, cell-free fetal DNA, which can be found in the plasma of pregnant women, also contributes to the phenomenon known as fetomaternal microchimerism. Already 25 years ago, the groundbreaking discovery of cell-free fetal DNA in the maternal plasma [121] has inspired the concept of non-invasive prenatal testing using maternal blood. Although this non-invasive approach already gained broad clinical acceptance for the detection of common fetal aneuploidies, it has still not reached the diagnostic level so far owing to the occurrence of a significant rate of false results [122, 123]. Since the cellular origin of cell-free fetal DNA in the mother is still a matter of debate, it is obvious that further research on potential candidates, such as placenta- and amniotic fluid-derived stem cells, can have important implications for the ongoing expansion of the clinical applications of non-invasive prenatal testing [78, 79, 120–122].

## Multipotent fetal stem cells as new therapeutic tools for reproductive system diseases

### The qualification for clinical applications

Many features, which have been described above to be beneficial for the usage of multipotent fetal stem cells for basic research, also highlight them as promising candidates for the development of innovative therapeutic applications. Fetal stem cells are easy to acquire, are not associated with ethical concerns, and are not covered by strict legal constraints. Furthermore, these stem cell entities are not highly demanding with regard to their in vitro propagation, since they harbor the potential of self-renewal with a high proliferation rate. Accordingly, fetal stem cells represent an easy-to-obtain, easy-to-handle and perfectly scalable source for the generation of therapeutic products derived from a high quantity of cells. And most importantly, due to their eminent differentiation capacity many of the multipotent fetal stem cell types can be developed into cells of all three embryonic germ layers what makes them deployable in the context of a wide spectrum of human pathologies (Table 1) [15, 32, 38, 39].

In addition, several other features of the here discussed stem cell entities are of particular advantage with regard to their translation to the bedside: 1) Fetal stem cells can be banked for their utilization in autologous stem cell approaches later in life [62]. 2) They exhibit an euploid karyotype, are genetically stable, are not expected to harbor many acquired mutations, and are not tumorigenic. Apart from the formation of malign tumors including metastatic events, even the tendency of stem cells to form benign growths in vivo could cause undesirable side effects and could have deleterious consequences for the therapeutic outcome. AFSCs and AECs are broadly multipotent human stem cells for which it has been demonstrated, that they do not even induce the formation of benign teratomas upon injection into animals (Table 1) [17, 67]. 3) Fetal stem cell-derived transplants are considered to be well tolerated by the patients´ immune system, because these stem cell types exhibit low immunogenicity. As depicted in Table 1 (see also the literature cited in this table), with the exception of umbilical cord blood-derived stem cells, all here described fetal stem cells do not express MHC class II molecules (only in a few reports AECs have been reported to be weakly positive for MHC class II molecules). Here it is important to add, that the inherent tumorigenic potential and immunogenicity of ESCs and iPSCs are currently still considered major hurdles for their clinical utilization [5, 6]. 4) The therapeutic effects of stem cell-derived transplants can be based on their integration into diseased target tissues and the acquisition and exercise of cellular functions to restore normal tissue homeostasis. Nonetheless, in the context of regenerative processes, the paracrine effects of transplanted stem cell products on endogenous cells and tissues play an equally important role [8, 19, 124]. Although fetal stem cells have been shown to secrete microRNAs [125], the understanding of their role for paracrine effects is still in its infancy. However importantly, the up to date-synopsis of their protein secretomes presented in Table 1 strongly suggests multipotent fetal stem cells to exhibit broad paracrine effects. And indeed, a paracrine potential to control the behavior of adjacent cells has been demonstrated for AFSCs [82], AF-MSCs [126], UCB-MSCs [91, 127–130], UCB-HSCs [131, 132], and for multipotent stem cells derived from the placenta [133–135]. 5) Finally, the attempts to use stem cells as vehicles or mediators of therapeutic concepts are subsumed under the term “next-generation stem cell approaches”. Stem cells can deliver promoters of apoptosis, oncolytic viruses or prodrug-converting enzymes or they can serve as mediators of gene therapy approaches such as gene editing or transduction of exogenous genes [8]. Multipotent fetal stem cells have been demonstrated to be highly amenable to genetic modifications [17, 40, 82, 84–92] what underscores their usability in next-generation stem cell approaches.

Having this wide spectrum of relevant features in mind, it is not surprising that multipotent fetal stem cells already moved into the center of endeavors to establish safe and efficacious new therapeutic concepts [31, 33, 136].

### In vitro differentiation of multipotent fetal stem cells into germ cells

In the last decade, the prevalence of infertility has significantly increased in the western world. Today, about 8–15% of individuals of reproductive age willing to conceive are supposed to be infertile [110, 112]. Infertility is defined upon verification of a specific impairment of a person´s capacity to reproduce or of the failure to achieve a pregnancy after a period of 12 months of unprotected sexual intercourse [137]. Beside hypogonadotropic hypogonadism, other specific diseases, gonadotoxic anti-cancer therapies, infections, or lifestyle-related factors, which can affect the fertility of both genders, also discrete causes for male and female infertility exist. Male infertility is mostly due to testicular deficiency and post-testicular impairment, whereas female infertility can be caused by fallopian tubal defects, tumors or polyps in the uterus or cervix, endometriosis, premature ovarian failure, or polycystic ovary syndrome [138]. Currently, it is assumed that up to 39% of infertility cases are related to male causes [138, 139]. One cause is non-obstructive azoospermia characterized by the absence of spermatozoa in the ejaculate. Beside idiopathic cases, the vast majority of these irreversible defects in spermatogenesis are the consequence of inflammatory, endocrine, or genetic disorders [108, 140]. An already existing approach to obtain biological offsprings is composed by sperm extraction upon testicular biopsy and intracytoplasmatic sperm injection. However, this strategy suffers significant limitations such as a low probability to find sperm cells and a low fertilization rate. In total, in the course of such attempts, the fertilization probability is 10–15% [112, 141, 142]. Since neither non-obstructive azoospermia nor, e.g., premature ovarian failure respond to drug therapy, adoption or the usage of donated sperms or eggs for in vitro fertilization are commonly chosen options. Building on the success of the research on human reproduction, assisted reproduction technologies have blossomed into widely and frequently used therapeutic instruments for infertility. However, the spectrum of currently available technologies cannot offer help for individuals, who do not develop functional gametes because of non-obstructive azoospermia or ovarian insufficiency, to conceive genetically related children [138, 143].

At present, two different strategies using stem cells for infertility treatment are pursued: the transplantation of stem cells or stem cell-derived paracrine factors to restore reproductive organ functions, which is discussed in the next chapter, and the in vitro differentiation of stem cells into germ cells or gametes [109, 110, 112].

During early embryonic development, pluripotent cells develop into PGCs, which then colonize the fetal gonads. These PGCs proliferate in the ovary as oogonia and receive signals from the adjacent somatic granulosa cells to differentiate into primary oocytes pausing at meiotic prophase. Finally, the hormone-driven maturation of oocytes starts in puberty. In the testis, proliferating gonocytes are surrounded by somatic Sertoli cells forming seminiferous tubules. Paracrine signals from Sertoli cells induce the differentiation of gonocytes into mitotically arrested prospermatogonia, which then differentiate into spermatogonial stem cells or spermatogonia after birth. Starting from puberty, the process of spermatogenesis is characterized by the transformation of mitotic stem cells into haploid gametes, designated spermatozoa. In summary, granulosa cells and Sertoli cells surrounding oogonia and gonocytes, respectively, together with the ovarian and testicular environment are of utmost importance for the development of female and male germ cells (Fig. 4). As a consequence of ovulation and fertilization with a spermatozoon, the oocyte completes the first and second meiotic divisions, respectively, to form the totipotent zygote (Figs. 1 and 4) [111, 144, 145].

**Fig. 5:**
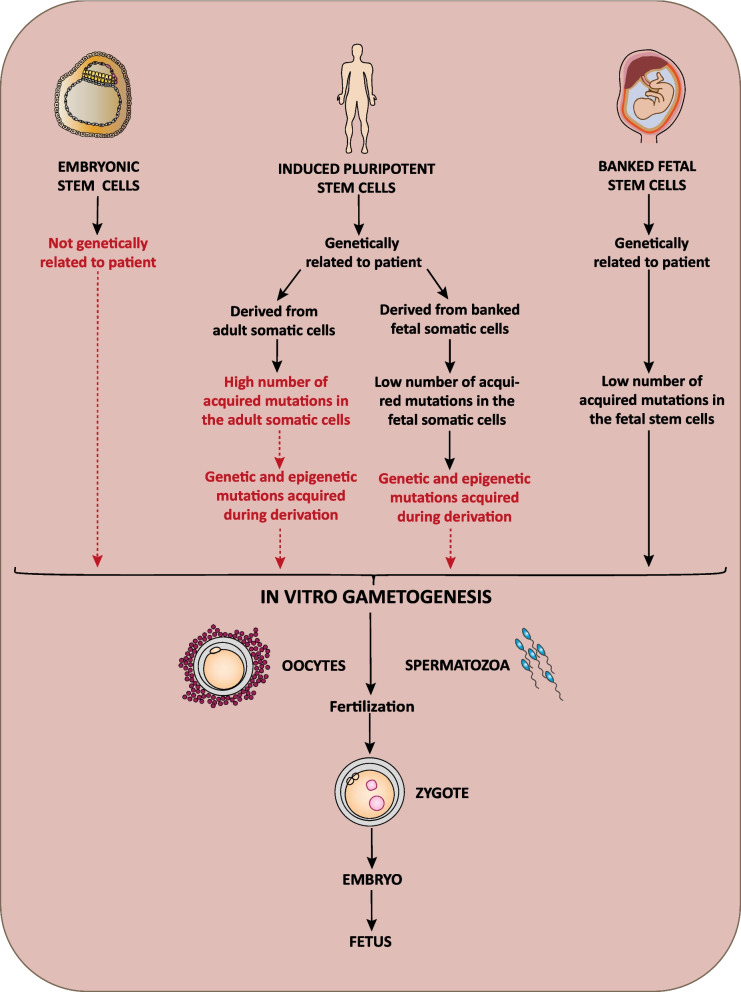
Stem cell-derived in vitro gametogenesis. Schematic comparison of the in vitro strategies to develop germ cells and gametes from embryonic stem cells, induced pluripotent stem cells, and banked fetal stem cells. For details see the text

With regard to in vitro germ cell development and gametogenesis, one currently pursued strategy includes the use of pluripotent stem cells. For a putative future application of so developed human gametes for assisted reproduction, only iPSCs generated from somatic cells of the advice-seeking individual but not ESCs would allow to produce genetically related children. Theoretically, ESCs-derived gametes could also be genetically related to parents when the ESCs are derived from an embryo generated from parental gametes. However, to treat infertility caused by the absence of functional gametes, these parental gametes would then still have to be developed from, e.g., iPSCs (Fig. 5). Whereas human pluripotent stem cells could only be developed into early oocytes and prospermatogonia so far, in vitro gametogenesis using murine pluripotent stem cells was already successful in inducing functional oocytes and spermatozoa [111]. Without doubt, these experimental approaches will form the basis for a more comprehensive understanding of the development of germ cells and gametes. However, the utilization of iPSCs is always accompanied by the risk of a putative influence of their tumorigenicity and high number of genetic and epigenetic mutations on the so obtained results [5, 6]. This high number is considered to reflect both the mutations acquired in the course of their derivation process [146] and the widely accumulated mutations in the initially employed somatic cells [147, 148]. In general, germ cells have been demonstrated to harbor a mutation rate that is tenfold lower than the rate of somatic cells [149]. Accordingly, it does not seem surprising that many mouse embryos derived from in vitro-generated oocytes died upon abnormal prenatal development and that the surviving animals tend to harbor anomalies [150, 151]. In order to circumvent this problem, the approach to generate iPSCs from fetal somatic cells, which are supposed to harbor fewer acquired mutations, has been suggested [111]. Whereas banking of fetal cells would allow the generation of iPSCs and the utilization of in vitro-developed gametes to produce genetically related offsprings later in life, the problem of mutations acquired in the course of the iPSCs derivation process would still remain (Fig. 5).

An attractive strategy to jump over both hurdles would be the direct in vitro differentiation of banked fetal stem cells into germ cells and gametes. This approach would allow to circumvent both problems, the high number of acquired mutations found in adult cells and the mutations manifesting during the process of iPSC derivation (Fig. 5). In addition, the epigenetic signature of specific fetal stem cells could probably provide a very appropriate starting point for in vitro gametogenesis. On the one hand, AFSCs, exhibiting a gene expression pattern similar to that of germ cells and PGCs, have been discussed to represent PGCs, which might have migrated from the tissue-specific microenvironment to the amniotic fluid [16, 42, 72]. And on the other hand, monkey and human PGCs have been demonstrated to originate from an amnion-like structure [152, 153]. Later in development the amnion becomes the innermost layer of the fetal membranes harboring two different types of the here discussed fetal stem cells, the AM-MSCs and the AECs (Figs. 2 and 3) [15, 34, 51–53]. Accordingly, it had probably even to be expected that AFSCs and amnion-derived AM-MSCs and AECs have been found to harbor the potential to differentiate into germ cells (Table 2) [154–159].

**Table 2:** Differentiation of multipotent fetal stem cells into germ cells

Stem cells	Differentiation	Germ cell sex	References
*Amniotic fluid*			
AFSCs	Follicular fluid triggered BMP15, ZP1, ZP2, and ZP3-positive oocyte-like cell differentiation	Female	[154]
	Germ cell maturation factors or follicular fluid induced the expression of germ cell markers	Female	[215]
	Follicular fluid induced the development of meiotic germ cells expressing markers for folliculogenesis and oogenesis	Female	[156]
AF-MSCs	Stem cells from the amniotic fluid without c-Kit selection were developed to embryoid bodies and proven to express PGC markers and markers of early germ cell development, including ACR, Dazl, Fragilis, Piwil2, RNf17, Stella, Stra8, and Vasa	Not applicable	[216]
*Amniotic membrane*			
AM-MSCs	Incubation with BMP4 and RA induced PGC/spermatogonia-like cells (positive for Dazl, Itgb1, Mvh, Piwil2, and Stra8)	Male	[157]
	BMP4 induced the differentiation of germ/oocyte-like cells positive for Oct4, SSEA4, Vasa, and the oocyte-related gene Gdf9	Female	[158]
	Induction of PGC markers upon treatment with RA (induction of c-Kit, SSEA4, Vasa; downregulation of Oct4)	Male	[159]
AECs	Medium containing serum substitute supplement triggered the development of oocyte-like cells expressing Dazl, Vasa, the oocyte-specific markers Gdf9 and ZP3, and the meiosis-specific markers DMC1 and SYCP3	Female	[155]
*Chorion laeve*			
CL-MSCs	BMP4 induced the differentiation of germ/oocyte-like cells positive for Oct4, SSEA4, Vasa, and the oocyte-related gene Gdf9	Female	[158]
*Wharton´s jelly*			
WJ-MSCs	RA/testosterone and testicular cell-conditioned medium induced CD49, c-Kit, Oct4, Stella, and Vasa-positive GCs	Male	[217]
	Cultivation with follicular fluid induced oocyte-like cells positive for Dazl, Oct4, Stra8, SYCP3, Vasa, ZP2, and ZP3	Female	[218]
	Figlα transfection/cultivation with follicular fluid induced oocyte-like cells positive for Dazl, Oct4, Stra8, Vasa, ZP2, and ZP3	Female	[219]
	BMP4 induced DMRT1, PLZF, Stra8, and SYCP3-positive GCs and some sperm-like cells	Male	[220]
	RA/testosterone and testicular-cell-conditioned medium induced germ cells positive for Dazl, SYCP3, and Vasa	Male	[221]
	BMP4/RA-mediated induction of PGC markers SSEA4, Stella, SYCP3, and Vasa	Male	[222]
	BMP4/RA and cultivation on amniotic epithelial and chorionic plate cells drove the development of GCs expressing Dazl, Fragilis, β1-integrin, α6-integrin, Oct4, Piwil2, PLZF, Stra8, and Vasa	Male	[223]
	Co-cultivation with placental cells induced germ/oocyte-like cells positive for Oct4 and Vasa and weakly positive for the oocyte markers Gdf9 and ZP3	Female	[224]
	Follicular fluid, FSH, LH, and estradiol induced oocyte-like cells positive for Gdf9, SYCP3, ZP1, ZP2, and ZP3	Female	[225]
	Co-cultivation with Sertoli cells induced GCs positive for Dazl, Stella, and Vasa	Male	[226]
	BMP4/RA and testicular and placental culture condition induced GCs positive for c-Kit, Dazl, Piwil2, and Vasa	Male	[227]
	Human WJ-MSCs differentiated into germ-like cells upon injection into mouse seminiferous tubules	Male	[161]
	CD61 overexpression and BMP4 triggered ACR, Prm1, Stra8, and SYCP3-positive GCs	Male	[228]
	RA/LIF/GDNF/putrescine/testosterone/FSH and Sertoli/Epididymal cell co-cultivation triggered the development of haploid spermatid-like cells positive for ACR, Dazl, ODF2, Prm1, and Vasa	Male	[160]
	BMP4 induced the differentiation of germ/oocyte-like cells positive for Oct4, SSEA4, Vasa, and the oocyte-related gene Gdf9	Female	[158]
	RA and Sertoli cell-conditioned medium induced GCs positive for Prm1 and Stra8	Male	[229]
	RA and Sertoli cell-conditioned medium triggered the differentiation of GCs with diminished Oct4 and PLZF expression and upregulated ACR, Prm1, Stra8, and SYCP3. Some secondary spermatocytes and spermatid-like cells developed	Male	[230]
	BMP4/RA and polarized or non-polarized red light irradiation induced Dazl, Fragilis, SYCP3, and Vasa expression	Male	[231]
	Follicular fluid and cumulus cells-conditioned medium triggered the development of oocyte-like cells positive for c-Kit, Gdf9, SYCP3, Vasa, ZP1, ZP2, and ZP3	Female	[232]
	Co-cultivation with testicular cells induced Fragilis, SYCP3, and Vasa-positive GCs	Male	[233]

AFSCs, c-Kit + amniotic fluid stem cells; AF-MSCs, amniotic fluid mesenchymal stem cells; AM-MSCs, amniotic membrane mesenchymal stem cells; AECs, amniotic epithelial cells; CL-MSCs, chorion laeve mesenchymal stem cells; WJ-MSCs, Wharton’s jelly mesenchymal stem cells; UCB-MSCs, umbilical cord blood mesenchymal stem cells; UCB-HSCs, umbilical cord blood hematopoietic stem cells; CP-MSCs, chorionic plate mesenchymal stem cells; CV-MSCs, chorionic villi mesenchymal stem cells

ACR, Acrosin; BMP, bone morphogenetic protein; CD, cluster of differentiation; CD61, also called integrin-β3; c-Kit, tyrosine-protein kinase Kit (receptor for SCF); Dazl, deleted in azoospermia like protein; DMC1, DNA meiotic recombinase 1; DMRT1, doublesex and Mab-3 related transcription factor 1; Figlα; folliculogenesis specific basic helix-loop-helix protein; Fragilis, an interferon-inducible gene coding for a transmembrane protein; FSH, follicle-stimulating hormone; GC, germ cell; Itgb1, integrin β-1; Gdf9, growth differentiation factor-9; GDNF, glial cell line-derived neurotrophic factor; LH, luteinizing hormone; LIF, leukemia inhibitory factor; Mvh, mouse Vasa homolog; Oct4, octamer-binding transcription factor 4; ODF2, outer dense fiber of sperm tails 2 protein; PGC, primordial germ cells; Piwil2, Piwi like RNA-mediated gene silencing 2 protein; PLZF, promyelocytic leukemia zinc finger; Prm1, protamine 1; RA, retinoic acid; Rnf17, ring finger protein 17; SSEA, stage specific embryonic antigen; Stella, also known as developmental pluripotency associated 3 protein; Stra8, stimulated by retinoic acid 8 protein; SYCP3, synaptonemal complex protein 3; Vasa, also designated DDX4 - DEAD-box helicase 4; ZP, zona pellucida sperm-binding protein or zona pellucida glycoprotein.

The overall underlying principle of in vitro germ cell differentiation is to subject multipotent fetal stem cells to conditions mimicking the ovarian or testicular environment. This can be achieved by co-culture with supportive cells, cultivation in cell-conditioned medium or, e.g., follicular fluid, incubation with germ cell induction/maturation factors such as, e.g., bone morphogenetic protein 4 (BMP4), retinoic acid (RA), testosterone, estradiol, follicle-stimulating hormone (FSH), luteinizing hormone (LH), or also, e.g., by transfection with the gene for the folliculogenesis specific basic helix-loop-helix protein (Figlα). In addition to the assessment of morphological features, successful germ cell differentiation is usually confirmed by the detection of specific markers such as Acrosin (ACR), the deleted in azoospermia like protein (Dazl), the interferon-inducible gene coding for the transmembrane protein Fragilis, growth differentiation factor-9 (Gdf9), outer dense fiber of sperm tails 2 protein (ODF2), Piwi like RNA-mediated gene silencing 2 protein (Piwil2), protamine 1 (Prm1), Stella (also known as developmental pluripotency associated 3 protein), the protein Stra8 stimulated by retinoic acid, the synaptonemal complex protein 3 (SYCP3), Vasa (also designated DDX4—DEAD-box helicase 4), or the zona pellucida sperm-binding proteins ZP1, ZP2, and ZP3. Using such approaches, a variety of different studies already demonstrated the successful development of multipotent stem cells derived from amniotic fluid, amniotic membrane, chorion leave, and Wharton´s jelly into female and male germ cells (Table 2 and references cited therein). Several of these studies also proved the detection of the haploid status in the course of the induced differentiation process [156, 160]. In addition, following another experimental strategy, in 2015 Chen et al. could show that human WJ-MSCs can differentiate into germ cells upon injection into mouse seminiferous tubules [161].

Taken together, the results obtained in the last years perfectly illustrate that fetal stem cells will play a pivotal role to obtain a more comprehensive picture of the underlying molecular processes of human germ cell development [108, 112]. The next foreseeable steps might include attempts to use murine fetal stem cells to generate functional and genetically stable gametes. Encouraged by the results already achieved with pluripotent stem cells [111, 150, 151] these gametes could be used for the in vitro-generation of mouse embryos. The spectrum of genetic and epigenetic mutations in the so obtained gametes and embryos can be compared to those derived from pluripotent stem cells focusing on their role for prenatally and postnatally detected anomalies. Although there is still a long way to go, this could represent a relevant first step towards putative future applications in human assisted reproduction (Fig. 5).

### Transplantation of multipotent fetal stem cells or their paracrine factors to restore reproductive organ functions

Beside in vitro gametogenesis, another currently emerging strategy to regain the chance for genetically related children in cases of azoospermia or premature ovarian failure is based on the idea to restore gametogenesis in vivo by the transplantation of stem cells or their paracrine factors. In the last years, very likely driven by the gain of knowledge regarding their high differentiation potential, low immunogenicity, and rich secretome, multipotent fetal stem cells have intensively been studied in this context. The most commonly used approach includes the transplantation of human fetal stem cells, their exosomes, microvesicles, or conditioned medium into rodent models of chemically induced azoospermia or premature ovarian failure. Interestingly, in experiments published, e.g., 2020 restoration of gonad functions was not only observed upon injection into testes or ovaries, but also in systemic application upon injection into the tail vein [162–165]. The standard evaluation of the curative process includes a detailed histological analysis of the quantity and quality of the gametes before and after stem cell treatment. Additionally, a variety of markers for germ cell differentiation and meiosis are examined together with indicators for proliferation, apoptosis, and anti-oxidative processes. Furthermore, the experimental outcome can be monitored by studying the hormonal status of the animals (Table 3). In 2019 it was reported that in animal models of premature ovarian failure the pregnancy rate after stem cell transplantation can be determined to ultimately demonstrate the beneficial effects [163, 166]. To put it in a nutshell, one has to inevitably come to the conclusion that the proof for the restorative potential of multipotent fetal stem cells is established. As can be gathered from Table 3 a high number of studies have convincingly shown that fetal stem cells of various origins definitely harbor the capacity to restore female and male gametogenesis in vivo.

**Table 3:** Multipotent fetal stem cells as therapeutic tools for infertility

Stem cells	Restoration strategy	Sex	References
*Amniotic fluid*			
AFSCs	Human AFSCs injected into busulfan-induced POF mice restored ovarian morphology and functions	Female	[215]
	Rat AFSCs mediated therapeutic effects on busulfan-induced azoospermia in rats	Male	[234]
AF-MSCs	Human AF-MSCs improved ovarian function in a physiological aging mouse model	Female	[235]
	Human AF-MSCs-derived exosomes exerted positive effects on ovarian granulosa cells in a mouse POF model	Female	[171]
*Amniotic membrane*			
AM-MSCs	Human AM-MSCs recovered ovarian function in a chemical-induced premature ovarian aging mouse model	Female	[236]
	Ultrasound-pretreated AM-MSCs transplantation increased reproductive organ weight and improved ovarian function in POI rats	Female	[237]
	Human AM-MSCs exerted a therapeutic activity in a natural ovarian aging mouse model (improving follicle numbers)	Female	[172]
	Human AM-MSCs injected into tail veins improved ovarian functions in a rat POI model	Female	[238]
	Human AM-MSCs recovered ovarian function in a mouse POF model	Female	[166]
	Human AM-MSCs transplanted into mouse testis upon busulfan-induced toxicity restored spermatogenesis	Male	[239]
	Tail vein or ovary injection of human AM-MSCs improved ovarian function in rats with chemotherapy-induced POI	Female	[165]
	Human AM-MSCs facilitated injured endometrial regeneration in a rat intrauterine adhesions model	Female	[178]
AECs	Human AECs recovered ovarian function in a chemical-induced premature ovarian aging mouse model	Female	[236]
	Injection of human AECs and AEC-conditioned medium into mouse ovaries protected against chemotherapy-induced damage	Female	[240]
	Human AEC-derived exosomes restored ovarian function in chemotherapy-induced POF mice by transferring microRNAs	Female	[241]
*Wharton´s jelly*			
WJ-MSCs	Injection of human WJ-MSCs into testis of chemically induced azoospermic mice induced murine germ cell differentiation	Male	[242]
	Human WJ-MSCs differentiated into germ-like cells upon injection into mouse seminiferous tubules	Male	[161]
	Intraperitoneal injection of WJ-MSCs mediated therapeutic effects on oviduct function and fertility in rats with salpingitis	Female	[175]
	Human WJ-MSCs recovered disturbed hormone secretion and folliculogenesis in a rat POF model	Female	[243]
	Tail vein-injected human WJ-MSCs improved the reserve function of perimenopausal rat ovaries via paracrine mechanisms	Female	[162]
	Transplantation of human WJ-MSCs in rabbits with chronic salpingitis partially restored fertility	Female	[244]
	Human WJ-MSCs-derived exosomes improved POI related to ovarian granulosa cell apoptosis caused by cisplatin chemotherapy	Female	[245]
	WJ-MSCs exhibited homing characteristics and migrated to injured oviducts in rabbit to promote epithelial cell growth	Female	[174]
	In a human phase I clinical trial intrauterine injection of WJ-MSCs increased the pregnancy rate in Asherman adhesion syndromes	Female	[179]
	Therapeutic effect of human WJ-MSCs on tubal factor infertility in a chronic salpingitis murine model	Female	[176]
	Microvesicles derived from human WJ-MSCs mediated therapeutic effects in a mouse POF model	Female	[246]
	Tail vein injection of human WJ-MSCs prevented chemotherapy-induced ovarian failure in rats	Female	[163]
	WJ-MSCs regulated ovarian stromal cell differentiation via TGFβ1 and repaired ovarian function in POI rats	Female	[168]
	Transplantation of human WJ-MSCs improved ovarian function in a rat model of autoimmune-induced POF	Female	[247]
	Extracellular vesicles derived from human WJ-MSCs recovered fertility of premature ovarian insufficiency mice	Female	[248]
	Tail vein-injected human WJ-MSCs repaired chemotherapy-induced POF in mice	Female	[164]
	Protective effects of human WJ-MSC-derived conditioned medium on a cisplatin-induced ovarian injury mouse model	Female	[249]
	Injection of human WJ-MSCs in the ovary tissue of POF rats increased the amount of ovarian follicles	Female	[250]
*Umbilical cord blood*			
UCB-MSCs	Injection of human UCB-MSCs into chemotherapeutic-induced azoospermic mice improved spermatogenesis	Male	[251]
	Human UCB-MSCs restored fertility in chemotherapy-induced POI mice	Female	[252]
	Administration of human UCB-MSCs improved degenerative changes in the follicles of CTX-induced POF mice	Female	[253]
*Placenta*			
CP-MSCs	3D-cultured human CP-MSC-spheroids enhanced ovarian function by inducing folliculogenesis	Female	[254]
	Transplanted human CP-MSCs restored ovarian function in chemotherapy-treated mice	Female	[255]
	Human CP-MSCs inhibited apoptosis of granulosa cells in autoimmune POF mice	Female	[256]
	CP-MSC-mediated antioxidant effects restored ovarian function in an ovariectomized rat model	Female	[257]
	Human CP-MSCs stimulated ovarian function in aged rats	Female	[169]
	EGF released from human CP-MSCs improved POI in a mouse model	Female	[170]
	Human CP-MSCs restored ovarian function and induced ovarian folliculogenesis in ovariectomized rats	Female	[167]
	Human CP-MSCs ameliorated chemotherapy-induced damage in mouse testis	Male	[258]
	Vascular remodeling by human CP-MSCs restored ovarian function in an ovariectomized rat model	Female	[173]
CV-MSCs	EGF released from human CV-MSCs improved POI in a mouse model	Female	[170]
	Human CV-MSCs ameliorated chemotherapy-induced damage in mouse testis	Male	[258]

AFSCs, c-Kit + amniotic fluid stem cells; AF-MSCs, amniotic fluid mesenchymal stem cells; AM-MSCs, amniotic membrane mesenchymal stem cells; AECs, amniotic epithelial cells; CL-MSCs, chorion laeve mesenchymal stem cells; WJ-MSCs, Wharton’s jelly mesenchymal stem cells; UCB-MSCs, umbilical cord blood mesenchymal stem cells; UCB-HSCs, umbilical cord blood hematopoietic stem cells; CP-MSCs, chorionic plate mesenchymal stem cells; CV-MSCs, chorionic villi mesenchymal stem cells

CTX, cyclophosphamide; POF, premature ovarian failure; POI, premature (primary) ovarian insufficiency; TGF, transforming growth factor

Although in the course of such approaches it has also been demonstrated that human fetal stem cells transplanted into murine gonads harbor the potential to differentiated into germ-like cells [161], evidence has been provided that the mechanisms underlying most of the detected improvements are paracrine. The discussed consequences of these paracrine effects include the reactivation of germ cell-specific gene expression, the induction of biochemical cascades driving gametogenesis and meiosis, the stimulation of angiogenesis and hormone production, and the reduction of oxidative stress, cellular senescence and apoptosis [108, 112] (see also the references cited in Table 3). Obviously, the diverse spectrum of factors secreted by fetal stem cells (Table 1) already suggested that their restorative potential could be composed of many different mediators. Although the according research is still in its infancy, some signalling cascades including the nerve growth factor (NGF)/tropomyosin receptor kinase (TrkA) pathway [163], the phosphoinositide 3-kinase (PI3K) pathway [167], the transforming growth factor β1 (TGFβ1)/SMAD3 pathway [168], the bone morphogenetic protein (BMP)/SMAD pathway [169], epidermal growth factor (EGF)-mediated nuclear factor erythroid 2-related factor 2 (NRF2) /heme oxygenase-1 (HO-1) activation [170], the forkhead-box-protein O3 (FOXO3) pathway [167], the miR-369-3p/YY1-associated factor 2 (YAF2)/programmed cell death 5 (PDCD5)/p53 pathway [171], as well as the function of the hepatocyte growth factor (HGF) [172], epidermal growth factor (EGF) [172], or vascular endothelial growth factor (VEGF) [173], have already been demonstrated to be involved in the here discussed fetal stem cell-mediated curative processes (see also Table 3).

Beside the reactivation of gametogenesis, multipotent fetal stem cells have also been shown to exhibit the capacity to address other pathological conditions playing a role in infertility. For example, upon systemic injection, WJ-MSCs have been demonstrated to migrate to injured rabbit oviducts to promote epithelial cell growth [174], or to trigger therapeutic effects on oviduct function and fertility in rats with salpingitis [175]. Another study reported that intravaginal inoculation of WJ-MSCs alleviated hydrosalpinx of the oviduct and improved the fertility in a chronic salpingitis murine model [176]. A specific cause of infertility is the Asherman syndrome determined by a severe damage of the endometrial basal layer as a consequence of a curettage or endometritis. In this condition scar tissue, fibrosis, and adhesions trigger intrauterine cavity obliteration leading to impaired fertility [177]. In 2022, using a rat intrauterine adhesion model, it was shown that intrauterine injection of human AM-MSCs combined with a scaffold material triggered endometrial regeneration, decreased the fibrosis areas, and increased the thickness of the endometrium, the number of endometrial glands, and the pregnancy rate [178]. However, already several years before, a phase I clinical trial demonstrated that the transplantation of WJ-MSCs on a collagen scaffold into the uterine cavity followed by an adhesion separation procedure could be used to successfully treat Asherman syndrome in humans. Without the detection of any adverse treatment-related events 26 patients became pregnant, of which eight delivered babies [179]. In summary, the already existing knowledge regarding the extensive variety of routes and mechanisms how multipotent fetal stem cells can encounter infertility emphasizes the importance of further detailed studies to pave the way to promising future clinical applications in humans.

Although so far their curative function for infertility is the best documented role of multipotent fetal stem cells in reproductive system diseases, first evidences for their relevance in the context of other conditions in reproduction have also already been provided. As described above, inadequate placentation caused by dysfunctional trophoblasts can trigger pregnancy-related pathologies, such as preeclampsia or intrauterine growth restriction [118, 180]. Several findings indicated that fetal stem cell-mediated paracrine effects can prompt dysfunctional trophoblast cells to reestablish their essential roles for placenta development. Performing in vitro and in vivo experiments, CP-MSCs were found to control proper trophoblast invasion and immune responses by inhibiting proinflammatory cytokines like interleukin-1β (IL-1β), tumor necrosis factor-α (TNF-α), and interferon-γ (IFN-γ) [134]. In addition, WJ-MSCs have been reported to control the proper function of trophoblasts [128, 181]. And finally, the transplantation of human WJ-MSCs into a lipopolysaccharide-induced rat preeclampsia model has been proven to revert the pathological symptoms [182].

Considering all these findings providing strong evidence for their curative potential, it is not surprising that a variety of clinical trials using multipotent fetal stem cells for female and male reproductive system diseases are currently under way or recruiting patients. In the context of female reproductive system disorders, WJ-MSCs are presently tested for their potential in the treatment of intrauterine adhesions, intraventricular hemorrhage, premature ovarian failure, thin endometrium, uterine scars, and other uterus injuries. Furthermore, WJ-MSCs are under investigation regarding their potential to treat erectile dysfunction (https://clinicaltrials.gov) [108]. Both clinicians and patients eagerly await the evaluation and publication of these trials. However, based on the already existing knowledge, it can be predicted with certainty that multipotent fetal stem cells are standing on the threshold to enter the clinical arena of reproductive system diseases.

## Conclusions

Multipotent fetal stem cells have advantages compared to adult stem cells with regard to the in vitro propagation and differentiation potential and compared to pluripotent stem cells regarding the occurrence of genetic and epigenetic mutations, tumorigenicity and immunogenicity. Fetal stem cells are perfectly qualified for the use in studies on conditions caused by the development of dysfunctional fetal cells and tissues and in approaches to obtain a more comprehensive picture of the consequences of fetomaternal microchimerism. Although these stem cells have been shown to harbor a potential to differentiate into germ cells, their development into fully functional gametes has not yet been demonstrated. In this context, it is relevant to highlight that before fetal stem cell-derived human gametes could once be used in assisted reproduction an extensive ethical discussion must be held. Obviously, such a discussion depends on the establishment of an in vitro protocol allowing the generation of gametes of unrestricted genetic and epigenetic integrity. It is interesting to speculate whether multipotent fetal stem cells could once represent a promising strategy to achieve this goal (Fig. 5). However, a probably even more promising approach to help individuals, who do not develop functional gametes, to conceive genetically related children, could be based on the paracrine capacities of in vivo transplanted multipotent fetal stem cells to restore reproductive organ functions. This potential has been proven in many independent animal studies. Furthermore, the first clinical trials in humans have already been initiated to test the paracrine therapeutic effects of multipotent fetal stem cells on infertility, but also, e.g., on preeclampsia. In conclusion, these stem cell entities will for sure fundamentally contribute to enhance the basic knowledge on human reproduction. But for their translation into the clinical work on reproductive system diseases, there are still several uncertainties and obstacles that need to be worked on. For the benefit of patients, the functionality, reproducibility, specificity, and safety of such stem cell-based therapeutic strategies must be investigated in detail. And there are still gaps in our knowledge regarding the origin, biological role, and characteristics of the different here discussed fetal stem cell types, that need to be closed, before they can find their way into everyday clinical practice.

## Data Availability

Not applicable.

## Abbreviations

ACR: Acrosin
AECs: Amniotic epithelial cells
AFSCs: C-Kit + amniotic fluid stem cells
AF-MSCs: Amniotic fluid mesenchymal stem cells
ALP: Alkaline phosphatase
AM-MSCs: Amniotic membrane mesenchymal stem cells
Ang: Angiopoietin
aFGF: Acidic fibroblast growth factor
bFGF: Basic fibroblast growth factor
BMP: Bone morphogenetic protein
CD: Cluster of differentiation
CD61: Also called integrin-β3
c-Kit: Tyrosine-protein kinase kit (receptor for SCF)
CL-MSCs: Chorion laeve mesenchymal stem cells
c-Myc: Cellular myelocytomatosis oncogene product
CP-MSCs: Chorionic plate mesenchymal stem cells
Cripto: Epidermal growth factor-like Cripto protein CR1
CTACK: Cutaneous T-cell-attracting chemokine
CTX: Cyclophosphamide
CV-MSCs: Chorionic villi mesenchymal stem cells
CXCR4: C-X-C Motif Chemokine Receptor 4, SDF-1-receptor
CXCL: C-X-C motif chemokine ligand
Dazl: Deleted in azoospermia like protein
DMC1: DNA meiotic recombinase 1
DMRT1: Doublesex and Mab-3 related transcription factor 1
EGF: Epidermal growth factor
EpSCs: Epithelial stem cells
ESCs: Embryonic stem cells
Figlα: Folliculogenesis specific basic helix-loop-helix protein
FOXC1: Forkhead box C1 protein
FOXO3: Forkhead-box-protein O3
Fragilis: An interferon-inducible gene coding for a transmembrane protein
FSH: Follicle-stimulating hormone
GATA: GATA-binding protein
GC: Germ cell
G-CSF: Granulocyte-colony stimulating factor
Gdf9: Growth differentiation factor-9
GDNF: Glial cell line-derived neurotrophic factor
GFAP: Glial fibrillary acidic protein
GM-CSF: Granulocyte macrophage-colony stimulating factor
HGF: Hepatocyte growth factor
HO-1: Heme oxygenase-1
HSCs: Hematopoietic stem cells
ICAM-1: Intercellular adhesion molecule-1
IGF: Insulin-like growth factor
IGFBP: Insulin-like growth factor-binding protein
IFN-γ: Interferon-γ
IL: Interleukin
IL-1β: Interleukin-1β
IL-1ra: Interleukin 1 receptor antagonist
Itgb1: Integrin β-1
iPSCs: Induced pluripotent stem cells
Klf4: Krüppel-like factor 4
LAP: Latency-associated peptide
LH: Luteinizing hormone
LIF: Leukemia inhibitory factor
MAP-2: Microtubule-associated protein-2
MBP: Myelin basic protein
MCP: Monocyte chemoattractant protein
MHC: Major histocompatibility complex
MIF: Macrophage migration inhibitory factor
MIP: Macrophage inflammatory proteins
MMP: Matrix metalloproteinase
MSCs: Mesenchymal stem cells
Mvh: Mouse Vasa homolog
Nanog: Homeobox protein Nanog
NRG1-B1: Neuregulin-1-B1
NGF: Nerve growth factor
NRF2: Nuclear factor erythroid 2-related factor 2
NSCs: Neural stem cells
Oct4: Octamer-binding transcription factor 4
ODF2: Outer dense fiber of sperm tails 2 protein
OPG: Osteoprotegerin
PAI-1: Plasminogen activator inhibitor-1
μPAR: Urokinase plasminogen activator receptor
PDCD5: Programmed cell death 5
PDGF: Platelet-derived growth factor
PGCs: Primordial germ cells
PGE2: Prostaglandin E2
PI3K: The phosphoinositide 3-kinase
Piwil2: Piwi like RNA-mediated gene silencing 2 protein
PLZF: Promyelocytic leukemia zinc finger
POF: Premature ovarian failure
POI: Premature (primary) ovarian insufficiency
Prm1: Protamine 1
RA: Retinoic acid
RANTES: Regulated on activation, normal T cell expressed and secreted = chemokine (C-C motif) ligand 5 (CCL5)
Rex1: Redox-sensing transcriptional repressor Rex1
Rnf17: Ring finger protein 17
SCF: Stem cell factor
SDF-1: Stromal cell-derived factor 1
Sox: SRY-box transcription factor
SSEA: Stage specific embryonic antigen
Stella: Also known as developmental pluripotency associated 3 protein
Stra8: Stimulated by retinoic acid 8 protein
SYCP3: Synaptonemal complex protein 3
Tβ4: Thymosin β4
TBX6: T-Box transcription factor 6
TARC: Thymus- and activation-regulated chemokine
TGF: Transforming growth factor
TIMP: Tissue inhibitor of metalloproteinases
TNF: Tumor necrosis factor
TNFR1: Tumor necrosis factor receptor 1
Tra-1-60 and Tra-1-81: Antibodies recognizing epitopes on podocalyxin
TrkA: Tropomyosin receptor kinase
UCB-HSCs: Umbilical cord blood hematopoietic stem cells
UCB-MSCs: Umbilical cord blood mesenchymal stem cells
Vasa: Also designated DDX4 - DEAD-box helicase 4
VCAM1: Vascular cell adhesion molecule 1
VEGF: Vascular endothelial growth factor
WJ-MSCs: Wharton’s jelly mesenchymal stem cells
YAF2: YY1-associated factor 2
ZP: Zona pellucida sperm-binding protein or zona pellucida glycoprotein
